# 

**Published:** 1918-08-31

**Authors:** 


RATES OF SUBSCRIPTION:

Twelve months, 10/10; Six months, 5/5; Three months, 2/9, post free to any address in the United Kingdom. Abroad, Twelve months, 13/-; Six months, 6/6; Three months, 3/3, post free. **THE HOSPITAL "Index" to Volume LXI., October 19 7 to March 1918, is now ready, price 3½d. per copy, post free.** Address The Manager, The Hospital, "The Hospital" Building, 28/29 Southampton Street, Strand, London, W.C. 2.

### The Rank Stripes of Ships' Surgeons.

The Board of Trade desire to point out that the standard uniform for the Mercantile Marine recommended by the Committee, whose report was recently issued as a Parliamentary Paper, Cd.9030, has not yet been officially authorised. If, and when, an Act of Parliament is passed giving statutory authority for the uniform, there will be an alteration in the rank stripes proposed by the Committee for ships' surgeons and pursers, to differentiate them from the stripes of naval medical and accountant officers; and to effect this it is proposed to insert a diamond in ships' surgeons' and pursers' stripes, as in the case of the stripes recommended by the Committee for chief, second, and third officers.

### Croix de Guerre for Ambulance Drivers.

The following voluntary ambulance drivers have been awarded the Croix de Guerre. They are members of the Convoi de l'Ecosse S.S.A. 20, and the awards are understood to have been earned upon the Somme and behind the Chemin des Dames. Lieutenant Middelton; Conducteurs Hope, Lee, Gordon, Black, Reid, White, Henry, Walls, Morton, Rowat, and Hallam, and the late Conducteurs Malcolmson, Lee, and Roper, who were killed. Mr. B. Hallam was wounded in rushing his ambulance with a last load through No Man's Land. This information has been received by the British Committee of the French Red Cross.

Lady Congreve, wife of General W. N. Congreve, V.C., has been awarded the French Croix de Guerre in recognition of her courage and coolness when the hospital area of Nancy, where she was nursing, was under shell fire and attacks from bombing aeroplanes.

### Passing Events and Topics.

### Islington's Tuberculosis Dispensary.

During the year ended April 30 last, 841 cases from the northern half of the Borough of Islington were treated in the Tuberculosis Department at the Great Northern Central Hospital, which was started by arrangement with the Islington Borough Council in May 1917. The attendances numbered 3,472. In addition, 1,510 visits were made to patients' homes, and a considerable number of contacts examined.

### Despite a South American Snow-storm.

In response to an appeal from Lord Northampton (chairman), the Great Northern Central Hospital has received a donation of £15 18s. 7d. from Mr. A. J. Avery, the Hon. Treasurer of the British Patriotic Committee, Quilmes (South America). The amount was collected at a lecture entitled "Tommy, the World's Wonder," by Mrs. David T. Herald.. The Hon. Treasurer writes: "On the night of the lecture it blew a gale and snowed hard, and, according to the papers, it had not previously snowed in Quilmes for eighty-eight years."

### German Hospital Patient's Revenge.

Apparently the severities of German hospital treatment are not reserved exclusively for enemy wounded. Hun methods of administration of any kind are not renowned for their sympathy and considerateness, and resentment on the part of those to whom they are applied is not uncommon. But a patient at a military hospital at Rosenheim has taken his revenge in an unusually effective way for the drastic treatment of nerve troubles which the Germans follow—an attempt to "make the punishment fit the crime," so to speak—by burning down the place. A Munich telegram to a Cologne newspaper states that the treatment which caused the act of incendiarism has led several times to revolt on the part of the patients.

